# Raman imaging of *Micrasterias*: new insights into shape formation

**DOI:** 10.1007/s00709-021-01685-3

**Published:** 2021-07-22

**Authors:** Martin Felhofer, Konrad Mayr, Ursula Lütz-Meindl, Notburga Gierlinger

**Affiliations:** 1 Department of Nanobiotechnology, University of Natural Resources and Life Sciences Vienna (BOKU), 1190 Vienna, Austria; 2 Department of Biosciences, University of Salzburg, 5020 Salzburg, Austria

**Keywords:** Confocal Raman microscopy, *Micrasterias*, Cell division, Cell wall, Pectin, Cellulose, Barite

## Abstract

The algae *Micrasterias* with its star-shaped cell pattern is a perfect unicellular model system to study morphogenesis. How the indentations are formed in the primary cell wall at exactly defined areas puzzled scientists for decades, and they searched for chemical differences in the primary wall of the extending tips compared to the resting indents. We now tackled the question by Raman imaging and scanned *in situ* *Micrasterias* cells at different stages of development. Thousands of Raman spectra were acquired from the mother cell and the developing semicell to calculate chemical images based on an algorithm finding the most different Raman spectra. Each of those spectra had characteristic Raman bands, which were assigned to molecular vibrations of BaSO_4_, proteins, lipids, starch, and plant cell wall carbohydrates. Visualizing the cell wall carbohydrates revealed a cell wall thickening at the indentations of the primary cell wall of the growing semicell and uniplanar orientation of the cellulose microfibrils to the cell surface in the secondary cell wall. Crystalline cellulose dominated in the secondary cell wall spectra, while in the primary cell wall spectra, also xyloglucan and pectin were reflected. Spectral differences between the indent and tip region of the primary cell wall were scarce, but a spectral mixing approach pointed to more cellulose fibrils deposited in the indent region. Therefore, we suggest that cell wall thickening together with a denser network of cellulose microfibrils stiffens the cell wall at the indent and induces different cell wall extensibility to shape the lobes.

**Supplementary Information:**

The online version contains supplementary material available at 10.1007/s00709-021-01685-3.

## Introduction

The question of how a plant cell achieves its shape is central for basic cell biological research, and applied sciences, as morphogenesis of a single cell finally determines the shape of tissues and organs. Numerous studies have therefore focused on genetic and cytoplasmic regulation of morphogenesis in different model systems. The algae *Micrasterias* with its two inversely arranged semicells forming a star-shaped, highly symmetric cell pattern has turned out to be a very appropriate model system for studying such processes (Lütz-Meindl 2016; Meindl 1993; Meindl and Kiermayer 1981). *Micrasterias* belongs to Streptophyta (or, in other words, to the streptohytan lineage of the Viridiplantae) and with its close genetic relationship to higher plants (Leliaert et al. 2012; Wodniok et al. 2011), a cell size of about 200 μm, and its uncomplicated cultivation (Schlösser 1982), it is a perfect plant-model system.

After mitosis, cell shape in *Micrasterias* forms simultaneously in the two developing semicells that arise from the separation of the old halves of the original cell by centripetal growth of a septum wall. While at the beginning of morphogenesis the young semicells represent undifferentiated flat bulbs, first indentations are formed about 75 min after mitosis by a sudden stop of growth in two exactly defined, symmetrically arranged areas at the primary cell wall (Meindl 1993). This leads to the formation of the first indentations that become the deepest ones at the final cell shape. This process of growth cessation at exactly defined areas is repeated several times during morphogenesis of *Micrasterias* until the final shape and size is reached, about 5 h after the onset of mitosis. At this point, the young semicell approximates a mirror-image of the old one with species-specific indentations of different depth and typical denticulated lobe tips. A rigid cellulose-rich secondary wall is deposited as soon as morphogenesis is completed and the primary wall is finally pushed off by a sudden onset of mucilage excretion through the cell wall pores (Lütz-Meindl 2016).

How this complex morphological differentiation of a single cell is achieved has puzzled scientists for decades. Three growth processes—tip growth, branching, and lobe broadening—interplay and result in the many different cell shapes in desmids (Lacalli 1975). In *Micrasterias*, already several players and regulators have been identified, but numerous questions are still unanswered. Early studies by Kiermayer (1964) have shown in turgor-reduced cells that a pre-pattern for morphogenesis of *Micrasterias* is present at the plasma membrane, indicating that the crucial steps for morphogenesis happen at the cell periphery and not inside the cell. This may be the plasma membrane carrying particular receptors for vesicle membranes that deliver cell wall material, on the one hand, and the cell wall itself with characteristic physical properties that allow an extension, on the other hand. Additionally, stage-specific switching of dictyosomes for the production of cell wall vesicles of different compositions has been suggested as the basis for morphogenesis (Meindl 1993; Meindl and Kiermayer 1981). The vesicle contents have been identified mainly as different combinations of low and highly esterified pectins, arabinogalactan proteins, and hemicelluloses by immuno-TEM methods (Brosch-Salomon et al. 1998; Eder and Lütz-Meindl 2008; Eder et al. 2008; Lutz-Meindl and Brosch-Salomon 2000). They deliver material to the growing wall and thus tailor the chemical composition of the cell wall and its physical abilities (Lütz-Meindl 2016).

TEM analysis of high-pressure frozen and cryo-substituted *Micrasterias* cells has shown that fusion of vesicles containing primary cell wall material occurs only in growing areas of developing cells but not at the indentations (Meindl et al. 1992). The vesicle fusion zones during growth are defined by local Ca^2+^ influx (Meindl 1982) and the actin cytoskeleton but not the microtubule system (Holzinger et al. 2002; Pflügl-Haill et al. 2000; Schmid and Meindl 1992). Any disturbance of the actin cytoskeleton or product formation at the dictyosomes leads to cell shape malformations. The fact that vesicles fuse not in the area of the indentations as soon as shaping starts has led to the suggestion that growing and non-growing wall segments may have different chemical and/or physical properties. These differences might affect the extensibility of the primary cell wall during growth and might thus represent the basis for morphogenesis (Lütz-Meindl 2016).

While biochemical, immuno-cytochemical studies and TEM-coupled electron energy loss spectroscopy (EELS) on *Micrasterias* cells have shown no differences in the chemical composition of growing and non-growing primary wall segments (Eder and Lütz-Meindl 2008; Eder et al. 2008), very early studies by Ueda and Yoshioka (1976) visualizedthicker layers of cellulose in the indentations when compared to the tips by Calcofluor staining. We now applied confocal Raman microscopy to gain better insights into the cell shaping process and the involvement of the cell wall.in.ing. Across the whole *Micrasterias* cell, Raman spectra were acquired and used for chemical imaging. This was possible as the detected inelastic scattering represents molecular vibrations, and thus, each spectrum at each pixel was a molecular fingerprint of *Micrasterias*. We scanned the old and developing semicells of dividing *Micrasterias*, and based on thousands of acquired Raman spectra, we imaged the distribution of organelles and cell constituents such as minerals, lipids, proteins, starch grains, and the primary and secondary cell walls. With multivariate spectral data analysis (unmixing and mixing approaches), we tackled the question whether chemical and/or structural changes in the cell wall might be responsible for the shaping of the lobes.

## Material methods

### Algal cultures

*Micrasterias* cells were cultivated in Erlenmeyer flasks in liquid Desmidiacean medium (Schlösser 1982) under semi-sterile conditions. Cells were kept at 20 ± 1 °C at a photoperiod of 14-h light:10-h dark. Cells were sub-cultured every 4–6 weeks, and 3- to 4-week-old cultures were used for experiments.

### Raman spectroscopy

*Micrasterias* cells were transferred on glass slides within a drop of water. A coverslip was carefully put on top and sealed with nail polish. In every prepared sample, we searched for different developmental stages to further acquire Raman spectra data sets using a confocal Raman microscope (Alpha300RA, WITec GmbH, Germany) equipped with a linear polarized VIS laser (λ_ex_ = 532 nm). The laser power was set to 20 mW and directed via a 100 × oil immersion objective (numerical aperture (NA) = 1.4, coverslip correction 0.17 mm; Carl Zeiss, Germany) onto the sample. The Raman signal was backscattered through the same objective, directed through an optic multifiber (50 μm diameter) to the spectrometer (600 g mm^−1^ grating; UHTS 300 WITec, Germany) and finally to the CCD camera (Andor DU401 BV, Belfast, North Ireland). The Control FOUR (WITec, Germany) acquisition software was used for the experimental setup. First, the area of interest was scanned very fast (0.01 s per px, 1-μm step) to bleach the chloroplast to minimize sample fluorescence in the subsequent scans. After this bleaching step, hyperspectral data sets were acquired from cells at different developmental stages by scanning the cells in 0.3-μm steps with 0.04-s integration time. Project FIVE Plus (WITec, Germany) was used for spectral processing and data analysis. After cosmic ray removal and baseline correction, the complete hyper spectral dataset was calculated as a linear combination of the most different spectra with a basis analysis algorithm (“true component analysis” in Project FIVE Plus WITec) (Dieing and Ibach 2011). By this, the main components were detected and their distribution visualized within the scanned area. To get detailed insights into the compositions of the cell wall, we modeled the spectra of the secondary and primary cell walls as a linear combination of measured reference carbohydrate spectra (Supplementary Table 1) using the Orthogonal Matching Pursuit (OMP) algorithm (Pati et al. 1993). For secondary cell wall, the cell wall spectra retrieved by true component analysis were analyzed, and for primary cell wall, average spectra were extracted by marking the extending tip and the indentations separately. Out of the many investigated cells and developmental stages, we exemplarily show one earlier and one later stage of development in this paper.

## Results

### Chemical images of developing Micrasterias cells

**Fig. 1:**
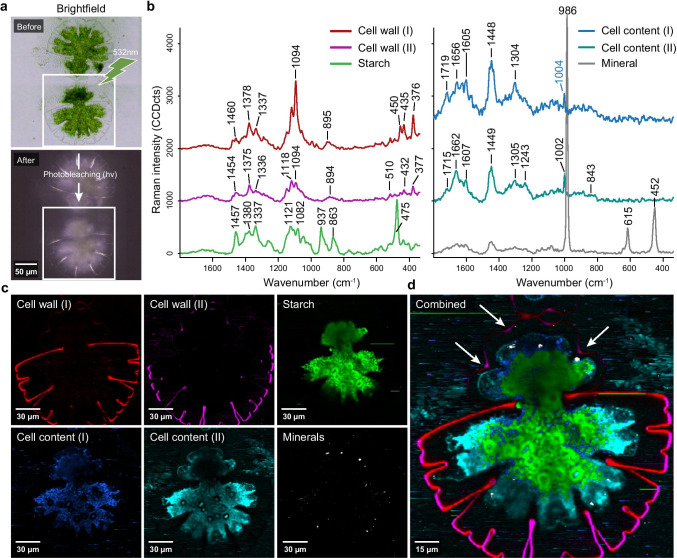
Raman imaging of *Micrasterias* showing the old and newly formed semicell in an early stage after cell division. **a** Light microscopic stitching images of the cell before (20 × objective) and after “bleaching” (100 × objective). A fast bleaching pre-run with green laser light discolored the chloroplast and minimized fluorescence background. **b** “True component analysis” (Witec5Plus) separated three different carbohydrate Raman spectra (red, pink green), two cell content spectra with mainly protein bands (blue, cyan), and one spectrum with sharp mineral bands attributed to BaSO_4_ (black) and visualized their **c** distribution in the old and developing semicell. **d** The combination image summarized the results and highlighted the strong cell wall signal in the old semicell, whereas in the developing semicell cell wall, material was only visualized in indentations (arrows)

The *Micrasterias* cells were embedded in water to minimize fluorescence. The cover slip was slightly pressed and sealed to avoid evaporation and ensure stable measurements. With this preparation, some cells were slightly squeezed, the protoplast moved back from the cell wall, and some cell contents were found outside the cell (Fig. 1). After a fast scan to bleach the chloroplast (Fig. 1a), high-quality spectra were obtained from cell contents and the cell wall (Fig. 1b, c). Applying true component analysis (@WitecPlus 5, (Dieing and Ibach 2011)) revealed three different carbohydrate spectra with strong bands at, e.g., 1093 cm^−1^, 1121 cm^−1^, 1378 cm^−1^, 376 cm^−1^, 937 cm^−1^, and 475 cm^−1^ (Fig. 1b, red, pink, green). Additionally, two different cell content spectra were derived with bands at, e.g., 1662 cm^−1^, 1448 cm^−1^, 1304 cm^−1^, 1004 cm^−1^ (Fig. 1b, blue, cyan), as had been found in proteins and lipids (Czamara et al. 2015; Rygula et al. 2013). Finally, one spectrum showed very sharp bands as typical for minerals (Fig. 1b). With bands at 986 cm^−1^, 615 cm^−1^, and 452 cm^−1^ (Fig. 1b, black line), it was assigned to barite (BaSO_4_), as exactly these three bands are reported as the strongest in BaSO_4_ (Zhou et al. 2020). The corresponding distribution maps showed that two of the carbohydrate spectra refer to the cell wall (red, pink) and one to the cell content (green) (Fig. 1c). The location around pyrenoids and the characteristic band at 475 cm^−1^ (Fig. 1b–d, green spectrum) pointed to starch. Comparison with a reference Raman spectrum of starch showed that all bands fitted well (Suppl. Figure 1) and thus confirmed that the green color component represented starch. Additionally, proteins and lipids filled up the cell and accumulated near the indentations of the developing semicell (Fig. 1c, blue, cyan). The barite crystals were detected in small clusters, mainly near the indents in the developing semicell (Fig. 1c, white). The combined distribution map visualized all components at once (Fig. 1d). The cell wall signal was strong in the mother cell, but the developing semicell showed almost no signal, except in the first indendations (Fig. 1d, arrows).

**Fig. 2:**
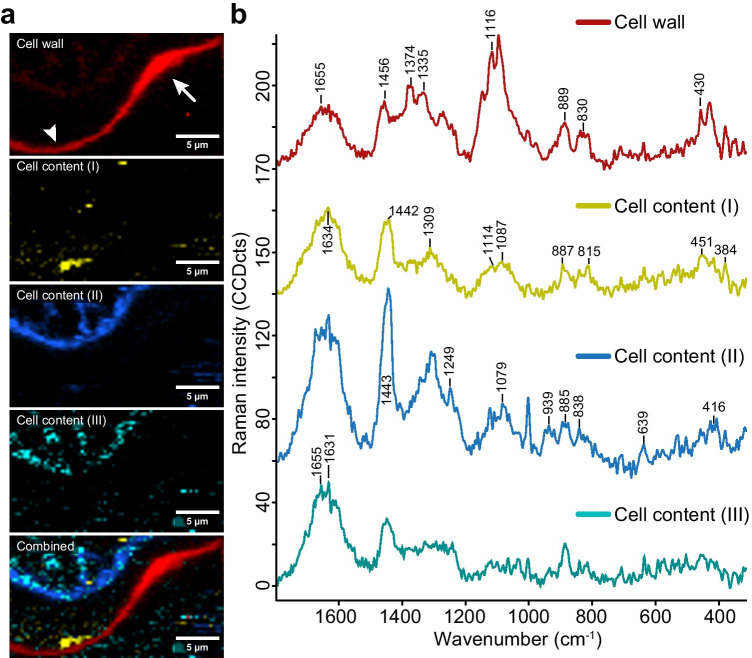
Raman imaging based on “true component analysis” revealed four different components in the developing semicell. **a** The cell wall (red) showed a thickening at the indent (arrow) whereas at the tip the cell wall was thin. Cell content below the tip (yellow) was separated from plasma membrane (blue) and vesicles (cyan). **b** The corresponding spectra confirmed the carbohydrate nature of the cell wall (red). The cell content below the tip showed bands attributed to protein, lipids, and carbohydrates/sugars (yellow). Spectra corresponding to plasma membrane and vesicles showed protein and lipid bands (blue and cyan)

Zooming into the cell wall of the developing semicell revealed details on protein distribution and cell wall deposition (Fig. 2a), one cell wall spectrum (red), one component below the lobe tip (yellow), and two other cell content spectra (blue, cyan, Fig. 2). No matter how many components have been set, there was only one cell wall spectrum retrieved. This means more or less the same cell wall composition throughout, but the cell wall thickness appeared again thicker in the indentation (arrow) compared to the regular thin walls of the lobes (arrowhead) (Fig. 2a, red). The cell content below the tip (yellow) displayed protein/lipid bands (1634 cm^−1^, 1442 cm^−1^) (Rygula et al. 2013), but some bands pointed also to carbohydrates (1114 cm^−1^, 1087 cm^−1^, 887 cm^−1^, 815 cm^−1^, 451 cm^−1^, 384 cm^−1^) (Fig. 2b). The other components displayed the protoplast (blue), and vesicles (cyan) and had typical protein bands, e.g., 1655, 1004 cm^−1^ (Fig. 2a, b).

**Fig. 3:**
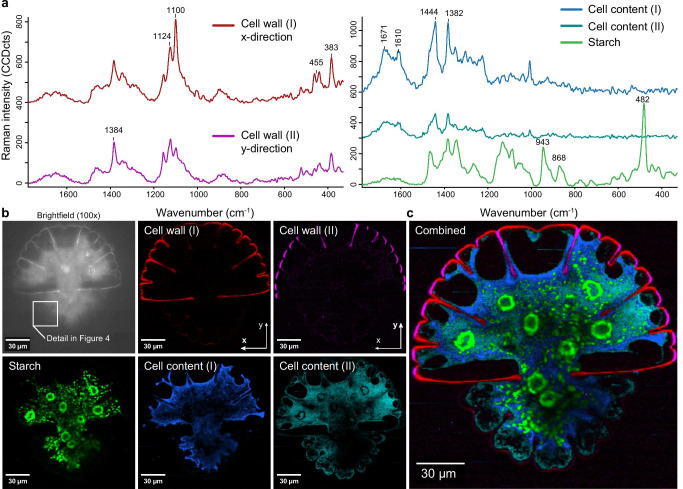
Raman imaging of *Micrasterias *showing the old and developing semicell in a later stage after cell division. **a** Five different spectra were retrieved by true component analysis: two cell wall spectra (red, pink), one starch spectrum (green) (Supplementary Fig. 1) and cell contents with protein and lipids (blue and cyan). The spectra are stacked with an offset of 400 and 300 CCDcts respectively for clarity. **b** Distribution of the components in the old and developing semicell: red cell wall was highlighted in x-direction, whereas the pink cell wall was attributed to cell wall in y-direction, starch highlighted the pyrenoids and smaller grains (green) and proteins and lipids (blue, cyan) were detected within the cell

In a later stage of development, similar component spectra were retrieved, but no minerals (Fig. 3a). Again, two cell wall spectra turned up, but this time it became more obvious that they might reflect the different orientation of the cellulose microfibrils: the red spectra were emphasized in the x-direction, while the pink components were mainly displayed in the y-direction (Fig. 3b, c). Also, in this stage, the Raman intensity in the younger primary cell walls was much lower than that in the secondary cell walls of the mother cell. Starch (green) and again protein and lipids (blue, cyan) filled out the cell and were at some places in contact with the cell wall, but mostly detached (Fig. 3b, c).

**Fig. 4:**
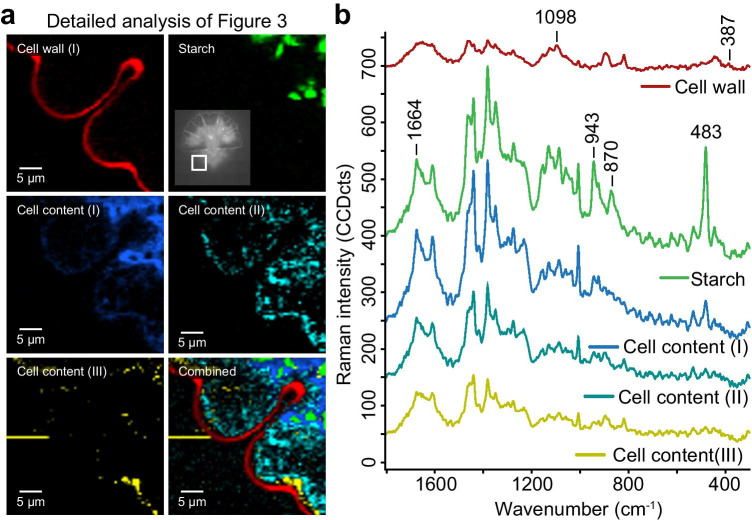
Raman imaging of the young semicell with one deep and one newly formed indentation. **a** Five different components were retrieved. Cell wall component (red) showed thickening in the oldest and deepest indent, but also in the newly formed less pronounced indent. One type of proteins/lipids (cyan) accumulated as vesicle below the cell wall, one kind (yellow) below the recently formed indent, and the rest towards the center (blue). **b** The corresponding spectra confirmed the carbohydrate nature of the cell wall (red), cell content with typical starch bands (green), and other cell contents with varying amounts of proteins and lipids (blue, cyan, yellow)

Detailed analysis of the developing cell confirmed again the thicker cell wall or accumulation of wall material in the indentations (Fig. 4a, b; red). Starch was visualized (green), as well as proteins and lipids within the cell (blue, cyan) and below the newly developed indent (yellow) (Fig. 4a, b).

### Raman signatures of Micrasterias cell walls

To get more insights into the composition of the *Micrasterias* cell walls, the retrieved component spectra were modeled as a linear combination of reference carbohydrate spectra using the Orthogonal Matching Pursuit (OMP) algorithm (Pati et al. 1993). The spectra library included cellulose spectra acquired with different laser polarization directions and various hemicelluloses and pectins (Suppl. Table 1).

**Fig. 5:**
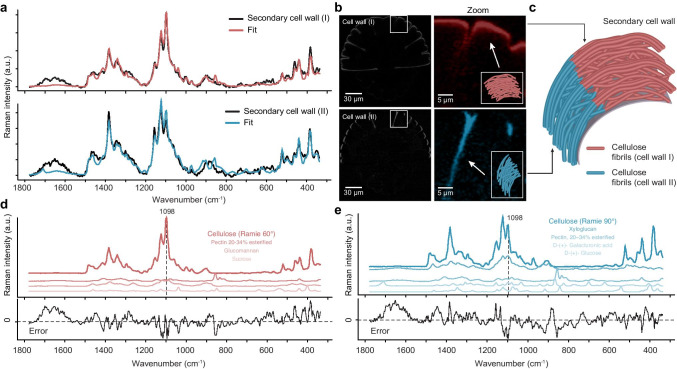
Raman spectra of secondary cell walls of *Micrasterias *were modeled as a linear combination of reference carbohydrate spectra using the Orthogonal Matching Pursuit method: (**a**) The cell wall spectra retrieved by true component analysis (see Fig. 3) were fit based on carbohydrate spectra. (**b**) The corresponding location in the *Micrasterias* cell was parallel (red) and perpendicular (blue) to the laser polarization direction (x-direction). (**c**) Model of the arrangement of cellulose fibrils in the secondary cell wall from the indent to the lobe. (**d**) Reference Raman spectra used to fit the cell wall derived in x-direction. Reference Raman spectra chosen for the secondary cell wall spectrum oriented perpendicularly to the laser polarization direction

Modeling the two distinguished cell wall spectra of the old mother cell (Figs. 3 and 5a–c) resulted in cellulose as the main contribution, but with a different orientation (Fig. 5d, e). While the first cell wall spectrum coincided best with Ramie (*Boehmeria nivea*) cellulose fiber acquired with 60° orientation with respect to the laser polarization (Fig. 5d), the other one reflected 90° orientation (Fig. 5e). The spectral changes in cellulose signature in secondary cell walls parallel and perpendicular to the laser polarization in the old mother cells pointed to highly ordered (crystalline) arrangement of the cellulose fibrils (Fig. 5c). Pectins and hemicelluloses were to a much lower extent reflected in the cell wall spectra (Fig. 5d, e).

**Fig. 6:**
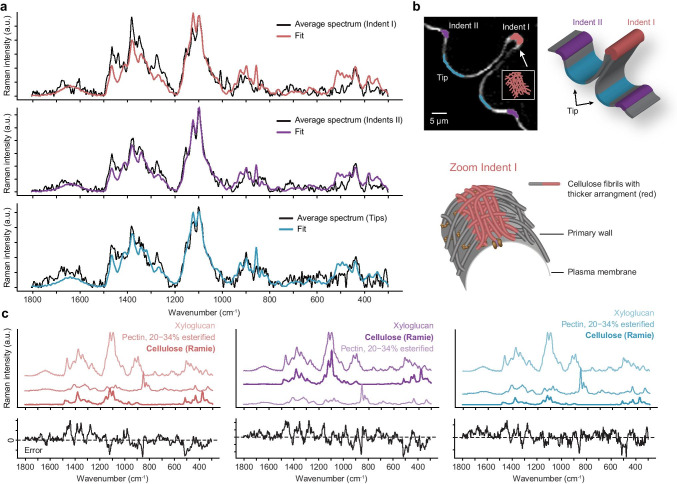
Average Raman spectra of primary cell walls of *Micrasterias *extracted from tip and indent regions were modeled as a linear combination of reference carbohydrate spectra using the Orthogonal Matching Pursuit method:** a** Fit of the average cell wall spectra extracted from indents and tip **b** as indicated in the scheme. At the indents, cell wall thickening was observed and **c** cellulose was fitted with more contribution in the indents than in the tips, while xyloglucan was the main contributor at all three positions

In contrast, the true component analysis of the primary cell wall of the developing semicell resulted in only one cell wall spectrum, so no orientation dependence and also no difference between tip and indent, at least based on the True component analysis (Figs. 2 and 4). To finally proof if there are no or at least minor spectral differences between the tip area and the indents, we marked these regions and extracted average spectra for OMP analysis (Fig. 6a, b). The spectra were noisier than secondary cell wall spectra and fitting revealed xyloglucan as the main contributor at all three positions, followed by pectin and/or cellulose (Fig. 6c). Although pectin with low esterification degree (20–34%) was chosen to fit into the primary cell wall at all three positions, there is a misfit at the pectin marker band at 852 cm^−1^ (Fig. 6a). This pectin marker band in primary cell walls of *Micrasterias* was much lower than in other primary cell walls of plants (e.g., Prats-Mateu et al. 2016). Spectra from the three positions were indeed very similar, but modeling gave an indication of slightly more cellulose in the thickened indent region than on the outgrowing tip (Fig. 6c).

## Discussion

Raman imaging on *Micrasterias* cells delivered a molecular fingerprint at every pixel. Scanning across the entire cell or regions of interest gave thousands of Raman spectra (hyperspectral data cube), which served to track all components at once. Cell wall polymers, starch, proteins, lipids, and barite were differentiated based on characteristic Raman spectra and their distribution followed within the mother cell and the growing semicell at two developmental stages (Figs. 1 and 3). No staining or reaction was necessary and thus accessibility and selectivity did not play a role. Without any fixation or drying, the cells were measured *in situ* in water—Raman imaging thus opened the view on the native state. With a resolution of about 300 nm, we resolved cell walls, protein/lipid vesicles, starch grains, and mineral crystals (Figs. 1, 2, 3, and 4).

### Straightforward mineral detection with micron-resolution and in context with the cell structure

The *in situ* capability of Raman microspectroscopy gives the unique possibility to study minerals within plant tissues (e.g., Dietrich et al. 2002; Gierlinger et al. 2008; Joester et al. 2017; Soukup et al. 2017; Weigend et al. 2018) and inside algae (e.g., Barcytė et al. 2020; Niedermeier et al. 2018). In addition to the chemical identity of minerals, Raman spectra are affected by crystal orientations (varying relative Raman band intensities), (sub)stoichiometric compositional changes (e.g., in solid solution series), traces of foreign ions, strain (the latter three shifting Raman bands), and crystallinity (changing Raman band widths), enabling comprehensive physicochemical characterization of minerals (Schmid and Dariz 2020). Based on the strong and sharp Raman bands at 986 cm^−1^, 615 cm^−1^, and 452 cm^−1^ (Fig. 1b, black line), we could identify barite (BaSO_4_) within the developing semicell of a dividing *Micrasterias* cell. Naturally occurring intracellular BaSO_4_ crystals have been reported in desmids (Meindl 1984; Wilcock et al. 1989) and other Zygnematophyceae (Brook et al. 1980; Kreger and Boere 1969). Barite crystals are typical for all of the Closterium species, but also for Pleurotaenium, Triploceras, and other similar genera (Brook 1981; Kreger and Boere 1969). In *Closterium moniliferum*, barite crystals were observed solely in terminal vacuoles (Wilcock et al. 1989), whereas in *Micrasterias*, BaSO_4_ crystals were found scattered throughout the whole cell (Meindl 1984) and seem to be a natural detoxification mechanism against the highly toxic barium (Niedermeier et al. 2018). In our study, the BaSO_4_ crystals accumulated near the plasma membrane at the indents in the developing semicell (Fig. 1). Earlier barium was shown to hyperpolarize the algal cell membrane and enhance plasmalemma resistance (Metlicka et al. 1996), and so might influence ion pumps and Ca^2+^ fluxes. Ca^2+^ plays a crucial role in the growth and development of dividing *Micrasterias* cells (Lehtonen and Volanto-Lumppio 1996), and is suggested for cell wall stiffening by binding with pectic polysaccharides (Eder et al. 2008). The Ca^2+^ cannot be detected with the Raman as we probe molecules and functional groups. Calcium would give strong Raman signals, e.g., as calcium oxalate crystal, which were not detected in this study. Only the barite was detected in the first sample near the indents. Barite was found in many of the investigated samples, but not in all and the shown pattern seems to be very stage and/or position (we probe only about 1 μm in depth) and/or sample-specific and would need further experimentations to draw clear conclusions.

### Cell wall spectra represent chemical composition and molecule orientation (crystallinity)

The strongest Raman signal was obtained in the secondary cell wall of the old mother cell. “True component analysis” revealed two cell wall variants and their distribution, one showed up in the x-direction (coinciding with laser polarization) and one more in the y-direction (Figs. 1 and 3, red and pink), pointed to an orientation effect of cellulose. Detailed spectral analysis showed coincidence with the spectra acquired from Ramie fibers with 60° and 90° laser polarization (Fig. 5). Ramie (*Boehmeria nivea*) fibers stand out among plant fibers due to their high concentration of cellulose (> 80%) (Satyanarayana et al. 2009), high crystallinity, and excellent mechanical properties (Jose et al. 2017). Microfibrils are aligned parallel with respect to the fibers and already early Raman polarization studies on Ramie helped to assign cellulose bands to molecular vibrations (Wiley and Atalla 1987). The good fit of the *Micrasterias* secondary cell wall spectra with spectra acquired from the Ramie fiber with 60 and 90° polarization direction (Fig. 5) confirm (1) the highly crystalline nature of *Micrasterias* cellulose (2) in plane orientation of the microfibrils with respect to the cell surface and (3) low amount of other cell wall polymers. While most algae and bacteria have a mixture of Iα and a smaller fraction of Iβ cellulose (Atalla and Vanderhart 1984; Sugiyama et al. 1991), *Micrasterias* secondary cell wall was reported as Iβ cellulose (Huang et al. 2019; Kim et al. 1996). Therefore, *Micrasterias* cellulose is more similar to the crystalline cellulose produced by vascular plants as now again proven by the coincidence of the Raman spectra of *Micrasterias* secondary cell wall with the cell wall of Ramie fibers (Fig. 5). Vibrational sum-frequency generation spectroscopy revealed recently a larger domain size of crystalline cellulose in the secondary cell wall of the *Micrasterias* cell compared to the primary cell wall of the developing semicell (Huang et al. 2019). The polymorphic structure of cellulose is suggested to be governed by the geometric shape of cellulose synthase complexes (Delmer 1999; Huang et al. 2019; Tsekos 1999; Tsekos et al. 1999), cellulose-water (Kubicki et al. 2018; O’Neill,^2017^; Oehme et al. 2018; Srinivas et al. 2014), and/or cellulose-matrix polymer interactions (Hackney et al. 1994; Park et al. 2014; Whitney et al. 1995, 1999). Xyloglucan and mixed-linked glucans ((1–3, 1–4)-ß-d-glucans) have been detected in the secondary cell wall of *Micrasterias* (Eder et al. 2008). Based on the mixing analysis, also two different hemicelluloses, glucomannan and xyloglucan, have been fitted into the *Micrasterias* cell wall spectra (Fig. 5d, e). Glucomannan is “seen” more in cell walls in x-direction and xyloglucan more in the walls in y-direction. A compositional change is not reasonable, but spectral orientation affects and thus alignment of hemicelluloses with respect to the cellulose, as has been detected in wooden cell walls by infrared spectroscopy (Stevanic and Salmen 2009). Nevertheless, for detailed analysis of hemicelluloses in *Micrasterias*, the reference library needs to be extended, if possible with the carbohydrates extracted from algae. Hemicelluloses have in general less Raman scattering intensity than crystalline cellulose due to the more amorphous nature and some bands overlap with cellulose bands (Agarwal et al. 2015, 2010). Therefore, they are difficult to track and need additional analysis, like the proposed Orthogonal Matching Pursuit analysis (Figs. 5 and 6).

While the Raman spectrum of the secondary cell wall reflected mainly crystalline cellulose (Fig. 5), the fitting of the spectrum of the primary cell wall of the developing semicell showed that hemicellulose and pectin contributed more (Fig. 6). The fit of the primary cell wall (Fig. 5d) is not as perfect as for the secondary cell wall (Fig. 5a), due to the lower signal/noise ratio and the multicomponent nature. Besides, as already mentioned probably not exactly the right hemicellulose and pectin is available in our database (Supplementary Table 1) and the native state, orientation and interactions between the polymers might furthermore change the spectral signature. For example, not included in the database were arabinogalactan proteins, which were found along the plasma membrane of the non-growing semicell (Eder et al. 2008). As hemicellulose, xyloglucans were found in the growing primary wall of *Micrasterias* (Eder et al. 2008) and are confirmed in our Raman spectra (Fig. 6). Besides, pectin contributes to primary cell walls (Fig. 5g), but the marker band around 856 cm^−1^, which is usually strong in pectins, is weak and lies within a broad overlapping band envelope (Figs. 6, 2b, 4b). The wavenumber position of this band is sensitive for the degree of methyl esterification: 858 cm^−1^ was reported for a low methyl esterification degree compared to 842 cm^−1^ for a high methylation degree (Synytsya,^2003^). In apple ripening, the pectin composition changes and can be monitored with a shift from 852 to 845 cm^−1^ (Szymanska-Chargot et al. 2016). From the three pectin reference spectra with 3 different degrees of esterification (Supplementary Table 1), the best fit was achieved with the spectrum acquired from citrus pectin with low esterification (Fig. 6). Pectic polysaccharides were shown to be transported to the cell wall in a de-esterified form (Eder and Lütz-Meindl 2008; Lütz-Meindl and Brosch-Salomon 2000), to become methyl-esterified at the inner side of the developing primary wall (Lütz-Meindl and Brosch-Salomon 2000) and become again de-esterified after translocation towards the outer side of the wall (see also Lütz-Meindl and Brosch-Salomon (2000). Also, the detailed structure of pectins in desmids is not yet known; there are some indications that algae contain higher contents of galacturonic and glucuronic acid than higher plants (Popper and Fry 2003) and that homogalacturonan accumulation in cell walls of Zygnema sp. (Charophyta) increases desiccation resistance (Herburger et al. 2019). Galacturonic acid was fit into the secondary cell wall, although with very low signal contribution (Fig. 5d). As the pectin-rich primary wall is finally pushed off by a sudden onset of mucilage production through the secondary wall and its pores (for details, see Oertel et al. (2004)), the galacturonic acid might represent remnants of the primary cell wall.

### Cell shape formation: finally a matter of cellulose deposition?

Pectin de-esterification processes were suggested to be crucial for morphogenesis and growth of *Micrasterias* (Eder and Lütz-Meindl 2008), although there is so far no indication that the degree of esterification is different in the areas of the forming indentations in comparison to the zones of the outgrowing lobes (Lütz-Meindl, 2016). In Raman spectroscopy, the pectin marker band around 852 cm^−1^ is strong in pectin references (Synytsya et al. 2003), but also in primary plant cell walls (see, e.g., Prats Mateu et al. (2016)). In *Micrasterias*, this band was much weaker than expected as also seen in the misfit at this positions (Fig. 6). As this band is attributed to the α glycosidic linkage (Synytsya et al. 2003), could it be that the pectin is much more “loose” with only short backbone chains in *Micrasterias*? Also to finally enable rapid dissolution and push off the primary cell wall (Oertel et al. 2004).

In the developing semicell, a cell wall thickening is observed in the indents in the early dividing stage (Figs. 1 and 2), as well as in the later developmental stages (Figs. 3 and 4) by Raman imaging, which reminds on Calcofluor cellulose staining of an earlier study (Ueda and Yoshioka 1976). Based on the high fluorescence at the indents, they suggested that the cell wall was already considerably differentiated at this position or might contain some special substances (Ueda and Yoshioka 1976). Based on the Raman signature, we suggest that cell wall thickening might go hand in hand with more cellulose fibrils laid down (Fig. 6b) and both together will achieve a stiffening at the indent. With the reduced extensibility due to a denser microfibril network, the morphogenesis is fixed. Very early studies by Kiermayer (1964) have shown in turgor-reduced cells that a pre-pattern for morphogenesis of *Micrasterias* is present at the plasma membrane. Exactly, at the plasma membrane, cellulose synthase complexes move to synthesize cellulose microfibrils (Turner and Kumar 2018). In contrast, other cell wall material is transported via vesicles and their fusion was only observed in the growing tip areas, but not at the indentations (Meindl et al. 1992), where probably more cellulose synthase complexes make a denser network of cellulose fibrils. Kinetic control of morphogenesis, i.e., by a reaction diffusion mechanism, implicated also the cell membrane as the most probable site of pattern formation (Lacalli 1982; Lacalli and Harrison 1987). In a relatively simple model, the importance of calcium ions and “loosening” enzymes was suggested as important starting points in plant cell morphogenesis. Reshaping of the cell wall was suggested as a relaxational process, wherein turgor pressure deforms the wall while enzymes allow the wall elements to slowly assume these stressed forms as their permanent forms (Kam and Levine 1997). But still it remains difficult to pick up experimentally how long a patterning event takes and it is supposed that patterning and its morphological expression might not be neatly separated in plants, but interact continuously (Harrison 2010).

Beside in the unicellular algae, big steps forward have been made in understanding polylobate shape formation in interdigitated puzzle cells of the epidermis (Lin and Yang 2020) as well as in the 3D interlocked sclerenchyma cells in nutshells (Antreich et al. 2021, 2019). Also, in these systems, cell wall components (cellulose and pectin) change locally cell wall extensibility and are considered important factors during shape formation. For epidermal puzzle cells, first, de-methylated pectin increases stiffness at the future indent and by this leads to cell wall undulation associated with higher stressed regions. As a result, more microtubules align and more cellulose fibrils are laid down at the indent, which slows down expansion at this location during growth (Altartouri,^2019^; Bidhendi et al. 2019). In the walnut shell cells, multiple loops of cellulosic thickenings in cell walls were detected to act as stiff restrictions during cell growth and by this leading to the lobed cell shape (Antreich et al. 2021).

With this work, we add another example on how complex and dynamic plant cell walls are, especially during development. Cellulose and pectin are the two most important components of the cell wall to tune for stiffness and extensibility, respectively. It is not only the amount of these two polymers that is changing, but also composition and dedicated alignment as micro- and/or nanofibrils, which adds fine-tuning. In the future, the carbohydrate library will be extended to get better fits and more detailed insights into the composition of the plant cell walls based on the Raman spectra. In this study on *Micrasterias* as well as in the development of polylobate epidermal and nutshell cells, an interplay of cellulose and pectin (with changing amount, composition, and structure) is involved to achieve the non-uniform expansion and shaping of lobes. The plant cell wall is not only a wall, but a dynamic feature with properties changing during development as well as locally along and across the cell wall.

## Supplementary Information

Below is the link to the electronic supplementary material.

Supplementary file1 (PDF 582 KB)

## References

[CR1] Agarwal U, Ralph S, Reiner R, Baez C (2015) Probing crystallinity of never-dried wood cellulose with Raman spectroscopy. Cellulose 23:125–144 10.1007/s10570-015-0788-7

[CR2] Agarwal UP, Reiner RS, Ralph SA (2010). Cellulose I crystallinity determination using FT–Raman spectroscopy: univariate and multivariate methods. Cellulose.

[CR3] Altartouri B (2019). Pectin chemistry and cellulose crystallinity govern pavement cell morphogenesis in a multi-step mechanism. Plant Physiol.

[CR4] Antreich SJ, Xiao N, Huss JC, Gierlinger N (2021) A belt for the cell: cellulosic wall thickenings and their role in morphogenesis of the 3D puzzle cells in walnut shells. Journal of Experimental Botany 72(13):4744–4756. 10.1093/jxb/erab197 10.1093/jxb/erab197 PMC8219037 33963747

[CR5] Antreich SJ, Xiao N, Huss JC, Horbelt N, Eder M, Weinkamer R, Gierlinger N (2019). The puzzle of the walnut shell: a novel cell type with interlocked packing. Adv Sci.

[CR6] Atalla RH, Vanderhart DL (1984). Native cellulose: a composite of two distinct crystalline forms. Science.

[CR7] Barcytė D, Pilátová J, Mojzeš P, Nedbalová L (2020). The arctic Cylindrocystis (Zygnematophyceae, Streptophyta) green algae are genetically and morphologically diverse and exhibit effective accumulation of polyphosphate. J Phycol.

[CR8] Bidhendi AJ, Altartouri B, Gosselin FP, Geitmann A (2019). Mechanical stress initiates and sustains the morphogenesis of wavy leaf epidermal cells. Cell Rep.

[CR9] Brook A, Fotheringham A, Bradly J, Jenkins A (1980). Barium accumulation by desmids of the genus Closterium (Zygnemaphyceae). Brit Phycol J.

[CR10] Brook AJ (1981) The biology of desmids vol 16. Univ of California Press

[CR11] Brosch-Salomon S, Hoftberger M, Holzinger A, Lutz-Meindl U (1998). Ultrastructural localization of polysaccharides and N-acetyl-D-galactosamine in the secretory pathway of green algae (Desmidiaceae). J Exp Bot.

[CR12] Czamara K, Majzner K, Pacia MZ, Kochan K, Kaczor A, Baranska M (2015). Raman spectroscopy of lipids: a review. J Raman Spectros.

[CR13] Delmer DP (1999). Cellulose biosynthesis: exciting times for a difficult field of study. Annu Rev Plant Biol.

[CR14] Dieing T, Ibach W (2011) Software requirements and data analysis in confocal Raman microscopy. In: Dieing T, Hollricher O, Toporski J (eds) Confocal Raman microscopy. Springer Berlin Heidelberg, Berlin, Heidelberg, pp 61–89. doi:10.1007/978-3-642-12522-5_4

[CR15] Dietrich D, Hemeltjen S, Meyer N, Baucker E, Ruhle G, Wienhaus O, Marx G (2002). A new attempt to study biomineralised silica bodies in Dactylis glomerata L. Anal Bioanal Chem.

[CR16] Eder M, Lütz-Meindl U (2008). Pectin-like carbohydrates in the green alga Micrasterias characterized by cytochemical analysis and energy filtering TEM. J Microsc.

[CR17] Eder M, Tenhaken R, Driouich A, Lütz-Meindl U (2008). Occurrence and characterization of arabinogalactan-like proteins and hemicelluloses in Micrasterias (Streptophyta) (1). J Phycol.

[CR18] Gierlinger N, Sapei L, Paris O (2008). Insights into the chemical composition of Equisetum hyemale by high resolution. Raman Imaging Planta.

[CR19] Hackney J, Atalla R, VanderHart D (1994). Modification of crystallinity and crystalline structure of Acetobacter xylinum cellulose in the presence of water-soluble β-1, 4-linked polysaccharides: 13C-NMR evidence. Int J Biol Macromol.

[CR20] Harrison L (2010) Micrasterias, and computing patterning along with growth. In: Harrison LG (ed) The shaping of life: the generation of biological pattern. Cambridge University Press, Cambridge, pp 77–104. doi:10.1017/CBO9780511973970.008

[CR21] Herburger K, Xin A, Holzinger A (2019). Homogalacturonan accumulation in cell walls of the green alga Zygnema sp. (Charophyta) increases desiccation resistance. Front Plant Sci.

[CR22] Holzinger A, Monajembashi S, Greulich KO, Lütz-Meindl U (2002). Impairment of cytoskeleton-dependent vesicle and organelle translocation in green algae: combined use of a microfocused infrared laser as microbeam and optical tweezers. J Microsc.

[CR23] Huang S, Kiemle SN, Makarem M, Kim SH (2019). Correlation between crystalline cellulose structure and cellulose synthase complex shape: a spectroscopic study with unicellular freshwater alga Micrasterias. Cellulose.

[CR24] Joester M, Seifert S, Emmerling F, Kneipp J (2017). Physiological influence of silica on germinating pollen as shown by Raman spectroscopy. J Biophotonics.

[CR25] Jose S, Rajna S, Ghosh P (2017). Ramie fibre processing and value addition. Asian Journal of Textile.

[CR26] Kam R, Levine H (1997). Unicellular algal growth: a biomechanical approach to cell wall dynamics. Phys Rev Lett.

[CR27] Kiermayer O (1964). Untersuchungen über die Morphogenese und Zellwandbildung beiMicrasterias denticulata Bréb. Protoplasma.

[CR28] Kim N-H, Herth W, Vuong R, Chanzy H (1996). The cellulose system in the cell wall of Micrasterias. J Struct Biol.

[CR29] Kreger D, Boere H (1969). Some observations on barium sulphate in Spirogyra. Acta Botanica Neerlandica.

[CR30] Kubicki JD, Yang H, Sawada D, O’Neill H, Oehme D, Cosgrove D (2018). The shape of native plant cellulose microfibrils. Sci Rep.

[CR31] Lacalli T (1975). Morphogenesis in Micrasterias: II Patterns of Morphogenesis. Development.

[CR32] Lacalli T Reaction-diffusion models and desmid morphogenesis. In: Developmental Order: Its Origin and regulation. Symposium of the Society for Developmental Biology (USA) (1982) Alan R. Liss, Inc. NY, USA.

[CR33] Lacalli TC, Harrison LG (1987). Turing’s model and branching tip growth: relation of time and spatial scales in morphogenesis, with application to Micrasterias. Can J Bot.

[CR34] Lehtonen J, Volanto-Lumppio K (1996). Significance of Ca2+ and K+ in Micrasterias growth and morphogenesis. Plant Cell Physiol.

[CR35] Leliaert F, Smith DR, Moreau H, Herron MD, Verbruggen H, Delwiche CF, De Clerck O (2012). Phylogeny and molecular evolution of the green algae. Crit Rev Plant Sci.

[CR36] Lin W, Yang Z (2020). Unlocking the mechanisms behind the formation of interlocking pavement cells. Curr Opin Plant Biol.

[CR37] Lütz-Meindl U (2016). Micrasterias as a model system in plant cell biology. Front Plant Sci.

[CR38] Lütz-Meindl U, Brosch-Salomon S (2000). Cell wall secretion in the green alga Micrasterias. J Microsc.

[CR39] Meindl U (1982). Local accumulation of membrane-associated calcium according to cell pattern formation in Micrasterias denticulata, visualized by chlorotetracycline fluorescence. Protoplasma.

[CR40] Meindl U (1984) Nachweis von Bariumsulfat in den Kristallen kultivierter Zellen von *Micrasterias denticulata* mittels energiedispersiver Röntgenanalyse (EDAX). Phyton 24(2):273–276

[CR41] Meindl U (1993). Micrasterias cells as a model system for research on morphogenesis. Microbiol Rev.

[CR42] Meindl U, Kiermayer O (1981). Bioassay for the determination of the anti-microtubule action of various compounds with the green-alga Micrasterias-Denticulata. Mikroskopie.

[CR43] Meindl U, Lancelle S, Hepler P (1992). Vesicle production and fusion during lobe formation in Micrasterias visualized by high-pressure freeze fixation. Protoplasma.

[CR44] Metlicka R, Rauferová L, Sigler K, Janacek K (1996). Ba^ 2^+ ions hyperpolarize algal cell membrane and enhance plasmalemma resistance without affecting ion and water contents. Gen Physiol Biophys.

[CR45] Niedermeier M, Gierlinger N, Lütz-Meindl U (2018). Biomineralization of strontium and barium contributes to detoxification in the freshwater alga Micrasterias. J Plant Physiol.

[CR46] O’Neill H (2017). Dynamics of water bound to crystalline cellulose. Sci Rep.

[CR47] Oehme DP, Yang H, Kubicki JD (2018). An evaluation of the structures of cellulose generated by the CHARMM force field: comparisons to in planta cellulose. Cellulose.

[CR48] Oertel A, Aichinger N, Hochreiter R, Thalhamer J, Lütz-Meindl U (2004). Analysis of Mucilage secretion and exretion in Micrasterias (Chlorophyta) by means of Immunoelectron microscopy and digital time lapse video microscopy1. J Phycol.

[CR49] Park YB, Lee CM, Kafle K, Park S, Cosgrove DJ, Kim SH (2014). Effects of plant cell wall matrix polysaccharides on bacterial cellulose structure studied with vibrational sum frequency generation spectroscopy and x-ray diffraction. Biomacromolecules.

[CR50] Pati YC, Rezaiifar R, Krishnaprasad PS Orthogonal matching pursuit: recursive function approximation with applications to wavelet decomposition. In: Proceedings of 27th Asilomar conference on signals, systems and computers, 1993. IEEE, pp 40–44. 10.1109/ACSSC.1993.342465

[CR51] Pflügl-Haill M, Vidali L, Vos JW, Hepler PK, Lütz-Meindl U (2000). Changes of the actin filament system in the green alga Micrasterias denticulata induced by different cytoskeleton inhibitors. Protoplasma.

[CR52] Popper ZA, Fry SC (2003). Primary cell wall composition of bryophytes and charophytes. Ann Bot.

[CR53] Prats Mateu B, Hauser MT, Heredia A, Gierlinger N (2016). Waterproofing in Arabidopsis: following phenolics and lipids in situ by confocal Raman microscopy. Front Chem.

[CR54] Rygula A, Majzner K, Marzec KM, Kaczor A, Pilarczyk M, Baranska M (2013). Raman spectroscopy of proteins: a review. J Raman Spectrosc.

[CR55] Satyanarayana KG, Arizaga GG, Wypych F (2009). Biodegradable composites based on lignocellulosic fibers—an overview. Prog Polym Sci.

[CR56] Schlösser UG (1982). Sammlung von algenkulturen. Berichte Der Deutschen Botanischen Gesellschaft.

[CR57] Schmid T, Dariz P (2020) Editorial for the Special Issue “Modern Raman Spectroscopy of Minerals”. Minerals 10(10):860. 10.3390/Min10100860

[CR58] Schmid V, Meindl U (1992). Microtubules do not control orientation of secondary cell wall microfibril deposition in Micrasterias. Protoplasma.

[CR59] Soukup M, Martinka M, Bosnic D, Caplovicova M, Elbaum R, Lux A (2017). Formation of silica aggregates in sorghum root endodermis is predetermined by cell wall architecture and development. Ann Bot.

[CR60] Srinivas G, Cheng X, Smith JC (2014). Coarse-grain model for natural cellulose fibrils in explicit water. J Phys Chem B.

[CR61] Stevanic JS, Salmen L (2009). Orientation of the wood polymers in the cell wall of spruce wood fibres. Holzforschung.

[CR62] Sugiyama J, Vuong R, Chanzy H (1991). Electron diffraction study on the two crystalline phases occurring in native cellulose from an algal cell wall. Macromolecules.

[CR63] Synytsya A, Čopíková J,  Matějka P,  Machovič, V (2003). Fourier transform Raman and infrared spectroscopy of pectins. Carbohydrate Polymers.

[CR64] Szymanska-Chargot M, Chylinska M, Pieczywek PM, Rosch P, Schmitt M, Popp J, Zdunek A (2016). Raman imaging of changes in the polysaccharides distribution in the cell wall during apple fruit development and senescence. Planta.

[CR65] Tsekos I (1999). The sites of cellulose synthesis in algae: diversity and evolution of cellulose-synthesizing enzyme complexes. J Phycol.

[CR66] Tsekos I, Orologas N, Herth W (1999). Cellulose microfibril assembly and orientation in some bangiophyte red algae: relationship between synthesizing terminal complexes and microfibril structure, shape, and dimensions. Phycologia.

[CR67] Turner S, Kumar M (2018). Cellulose synthase complex organization and cellulose microfibril structure. Philos Trans R Soc A Math Phys Eng Sci.

[CR68] Ueda K, Yoshioka S (1976). Cell wall development of Micrasterias americana, especially in isotonic and hypertonic solutions. J Cell Sci.

[CR69] Weigend M, Mustafa A, Ensikat HJ (2018). Calcium phosphate in plant trichomes: the overlooked biomineral. Planta.

[CR70] Whitney SE, Brigham JE, Darke AH, Reid JG, Gidley MJ (1995). In vitro assembly of cellulose/xyloglucan networks: ultrastructural and molecular aspects. Plant J.

[CR71] Whitney SE, Gothard MG, Mitchell JT, Gidley MJ (1999). Roles of cellulose and xyloglucan in determining the mechanical properties of primary plant cell walls. Plant Physiol.

[CR72] Wilcock J, Perry CC, Williams RJP, Brook A (1989). Biological minerals formed from strontium and barium sulphates. II. Crystallography and control of mineral morphology in desmids. Proc R Soc Lond B Biol Sci.

[CR73] Wiley JH, Atalla RH (1987). Band assignments in the Raman spectra of celluloses. Carbohydr Res.

[CR74] Wodniok S, Brinkmann H, Glöckner G, Heidel AJ, Philippe H, Melkonian M, Becker B (2011). Origin of land plants: do conjugating green algae hold the key?. BMC Evol Biol.

[CR75] Zhou L, Mernagh TP, Mo B, Wang L, Zhang S, Wang C (2020). Raman study of barite and celestine at various temperatures. Minerals.

