# Supplementary material for: Raman imaging of Micrasterias: new insights into shape formation
Source: Protoplasma. 2021 Jul 22;258(6):1323–34. doi: 10.1007/s00709-021-01685-3 (PMC8523415; doi:10.1007/s00709-021-01685-3)
Supplement: Supplementary file 1 — Supplementary file1 (PDF 582 KB) [file 709_2021_1685_MOESM1_ESM.pdf]

Supplementary Information for

Raman imaging of Micrasterias: new insights into shape formation

Martin Felhofer[1], Konrad Mayr[1], Ursula Lütz-Meindl[2], Notburga Gierlinger[1*]
[1]Department of Nanobiotechnology, University of Natural Resources and Life Sciences Vienna (BOKU), 1190 Vienna, Austria
[2] University of Salzburg, Department of Biosciences, 5020 Salzburg, Austria.

*Author for correspondence: burgi.gierlinger@boku.ac.at

Supplementary Table 1: Reference library used for mixture analysis

Compound	CAS	Embedding medium	Producer	Annotation
Arabinoxylan	9040-27-1	D_2O	Megazyme	from rye flour
Cellulose	9004-34-6			from Ramie, laser polarization 0°
Cellulose	9004-34-6			from Ramie, laser polarization 15°
Cellulose	9004-34-6			from Ramie, laser polarization 30°
Cellulose	9004-34-6			from Ramie, laser polarization 60°
Cellulose	9004-34-6			from Ramie, laser polarization 90°
Cellulose	9004-34-6			
Cellulose (Avicel)	9004-34-6	H_2O	Dupond	
Glucomannan	11078-31-2	H_2O	Megazyme	from konjac tubers
Xyloglucan	37294-28-3	H_2O	Megazyme	
D-(+)- Galacturonic acid	91510-62-2	D_2O	Sigma	monohydrate
D-(+)- Galacturonic acid	91510-62-2	H_2O	Sigma	monohydrate
D-(+)- Glucose	50-99-7		Sigma	
1,3- Glucan	9051-97-2		Sigma	
Sucrose	57-50-1	D_2O		
Polygalacturonic acid	25990-10-7		Sigma	
Pectin, 85% esterified	37251-70-0	H_2O	Sigma	from citrus fruit
Pectin, 55-70% esterified	37251-70-0	H_2O	Sigma	from citrus fruit
Pectin, 20-34% esterified	37251-70-0	H_2O	Sigma	from citrus fruit
Starch	9005-25-8	H_2O		from potato
Starch	9005-25-8	H_2O		from wheat

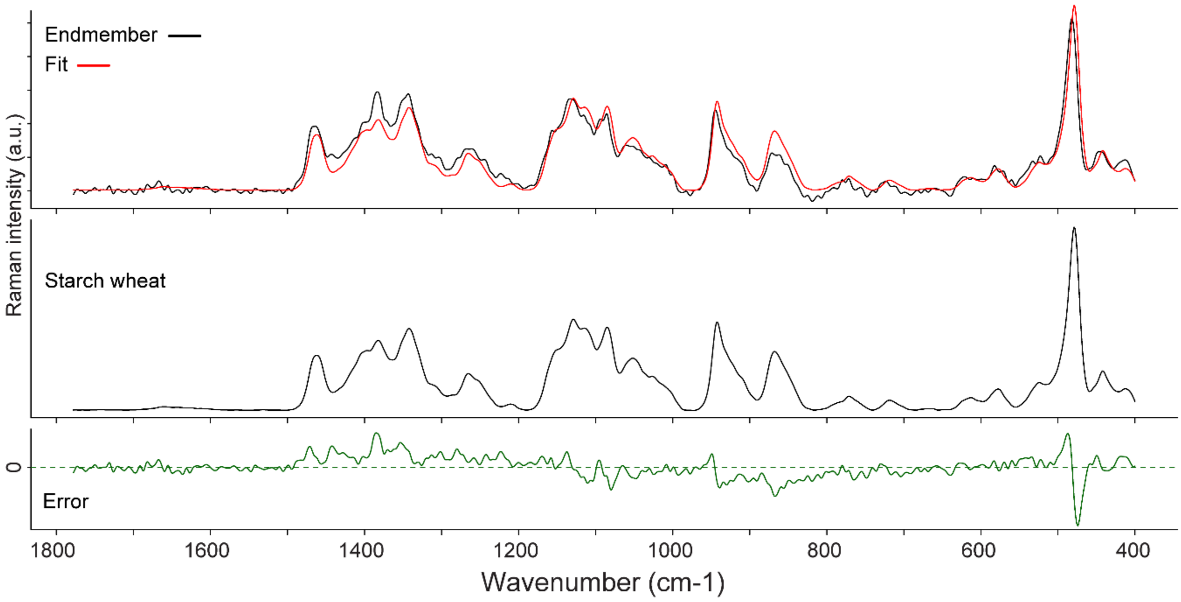

Supplementary Figure 1. Raman spectrum (black) from the true component analysis in Figure 3 main text showed coincidence with the starch reference spectrum and was thus the only spectrum chosen from the database (see Supplementary Table 1) for the fit (red).
